# Genetic resources for advanced biofuel production described with the Gene Ontology

**DOI:** 10.3389/fmicb.2014.00528

**Published:** 2014-10-10

**Authors:** Trudy Torto-Alalibo, Endang Purwantini, Jane Lomax, João C. Setubal, Biswarup Mukhopadhyay, Brett M. Tyler

**Affiliations:** ^1^Department of Biochemistry, Virginia Polytechnic Institute and State UniversityBlacksburg, VA, USA; ^2^Virginia Bioinformatics Institute, Virginia Polytechnic Institute and State UniversityBlacksburg, VA, USA; ^3^European Molecular Biology Laboratory, European Bioinformatics Institute (EMBL-EBI), Wellcome Trust Genome CampusCambridge, UK; ^4^Departamento de Bioquímica, Instituto de Química, Universidade de São PauloSão Paulo, Brazil; ^5^Department of Biological Sciences, Oregon State UniversityCorvallis, OR, USA; ^6^Center for Genome Research and Biocomputing, Oregon State UniversityCorvallis, OR, USA

**Keywords:** Gene Ontology, advanced biofuels, synthetic biology, cellulosome, advanced alcohols, fatty acid-derived fuel, isoprenoid-derived fuel

## Abstract

Dramatic increases in research in the area of microbial biofuel production coupled with high-throughput data generation on bioenergy-related microbes has led to a deluge of information in the scientific literature and in databases. Consolidating this information and making it easily accessible requires a unified vocabulary. The Gene Ontology (GO) fulfills that requirement, as it is a well-developed structured vocabulary that describes the activities and locations of gene products in a consistent manner across all kingdoms of life. The Microbial ENergy processes Gene Ontology () project is extending the GO to include new terms to describe microbial processes of interest to bioenergy production. Our effort has added over 600 bioenergy related terms to the Gene Ontology. These terms will aid in the comprehensive annotation of gene products from diverse energy-related microbial genomes. An area of microbial energy research that has received a lot of attention is microbial production of advanced biofuels. These include alcohols such as butanol, isopropanol, isobutanol, and fuels derived from fatty acids, isoprenoids, and polyhydroxyalkanoates. These fuels are superior to first generation biofuels (ethanol and biodiesel esterified from vegetable oil or animal fat), can be generated from non-food feedstock sources, can be used as supplements or substitutes for gasoline, diesel and jet fuels, and can be stored and distributed using existing infrastructure. Here we review the roles of genes associated with synthesis of advanced biofuels, and at the same time introduce the use of the GO to describe the functions of these genes in a standardized way.

## INTRODUCTION

**FIGURE 1:**
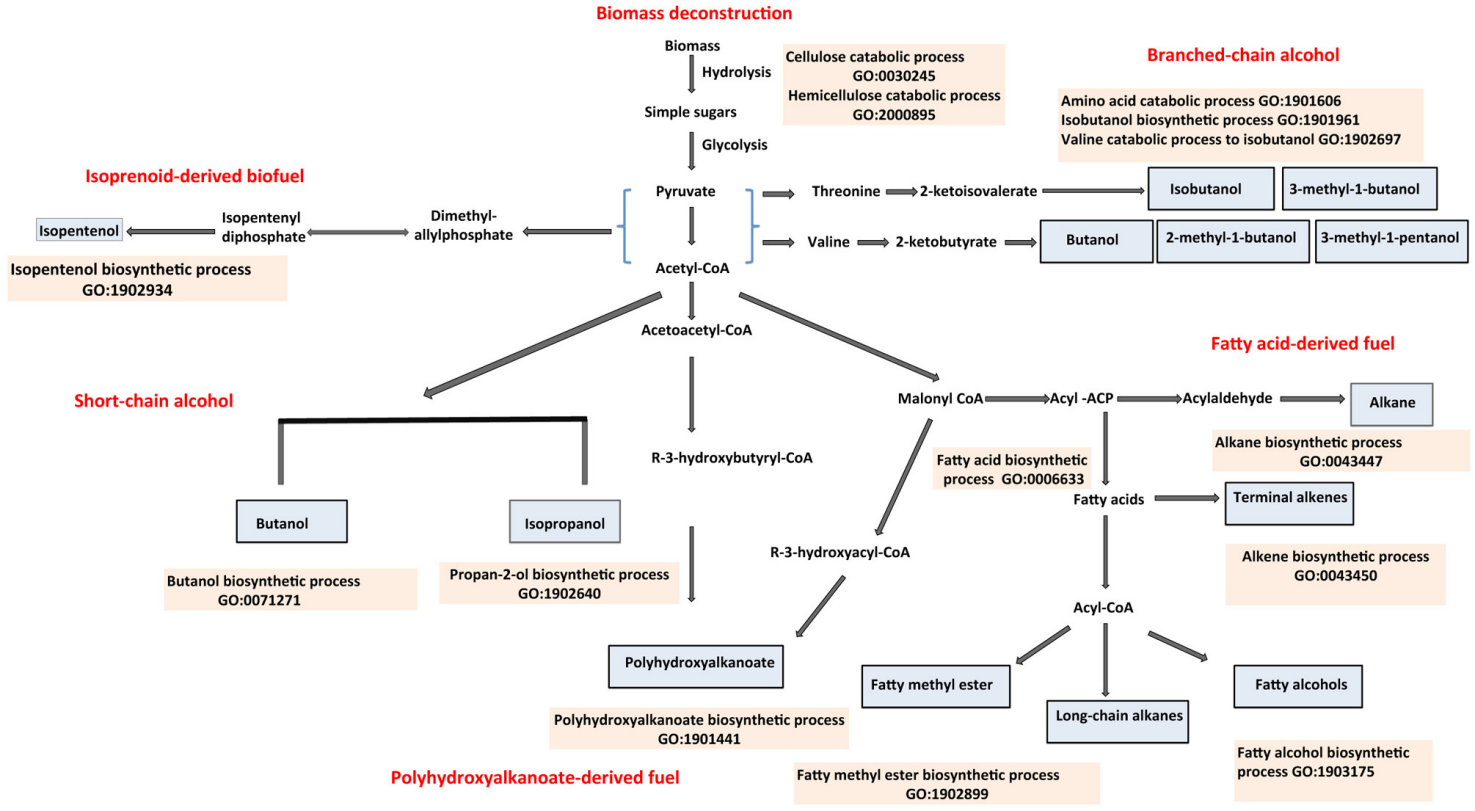
**Overview of the production of advanced biofuel precursors or products described with Gene Ontology (GO) terms.** Advanced alcohols such as isopropanol and butanol are synthesized via the CoA-dependent pathways; isobutanol, and other branched chain alcohols are produced via the keto-acid pathway and isopentenol via the isoprenoid pathway. Other advanced biofuel products, which include alkanes, alkenes and fatty methyl esters, are derived from fatty acid biosynthesis. Fuel products or their precursors are shown in sky blue shaded boxes. GO descriptions of the biological processes involved in the biosynthesis are shown in tan-shaded boxes. Relevant references are in the text.

Depletion of the world’s fossil fuel resources and environmental concerns associated with the emission of greenhouses gases has fueled interest in renewable and environmentally friendly alternatives (Köne and Büke, 2010; Hansen et al., 2013; Suranovic, 2013). In this context, advanced biofuels have been of growing interest as these compounds can be generated from non-food cellulosic biomass, can be added directly to gasoline or diesel or sometimes used as stand-alone fuel, and can be stored and distributed using existing infrastructure (Dürre, 2007; Mehta et al., 2010; Weber et al., 2010). Advanced biofuels include alcohols such as butanol, isopropanol, and isobutanol, and fuels derived from fatty acids, isoprenoids, and polyhydroxyalkanoates (PHAs). Advanced biofuels from lignocellulose feedstock begins with biomass deconstruction. The cellulosic component of the biomass is degraded into pentoses and hexoses. The multienzyme complexes involved in the degradation of cellulosic biomass are discussed in this review. The native and engineered pathways leading to the production of advanced biofuels have been studied extensively. As such the primary published literature is rich in information derived from research on these fuels but this information has yet to be aggregated in a manner that is easily accessible and amenable to computational analysis. The well-established Gene Ontology (GO; Ashburner et al., 2000) provides a basis for harnessing this information. The GO provides sets of standardized terms to describe molecular functions, biological processes and cellular components across all kingdoms of life (Ashburner et al., 2000; Harris et al., 2004; Torto-Alalibo et al., 2009; Dimmer et al., 2012). The Microbial ENergy processes Gene Ontology (MENGO) consortium^1^, an associate of the GO, initiated an effort in 2011 to extend the GO by developing missing terms associated with bioenergy processes. This effort has generated over 600 bioenergy-related GO terms, most of which are described in this review and the MENGO website^1^. These new terms, together with existing ones in the GO, were used to annotate gene products involved in bioenergy processes. Our emphasis is on selected native pathways leading to the generation of advanced biofuels. The scope of the review is summarized in **Figure 1**.

## THE GENE ONTOLOGY

**FIGURE 2:**
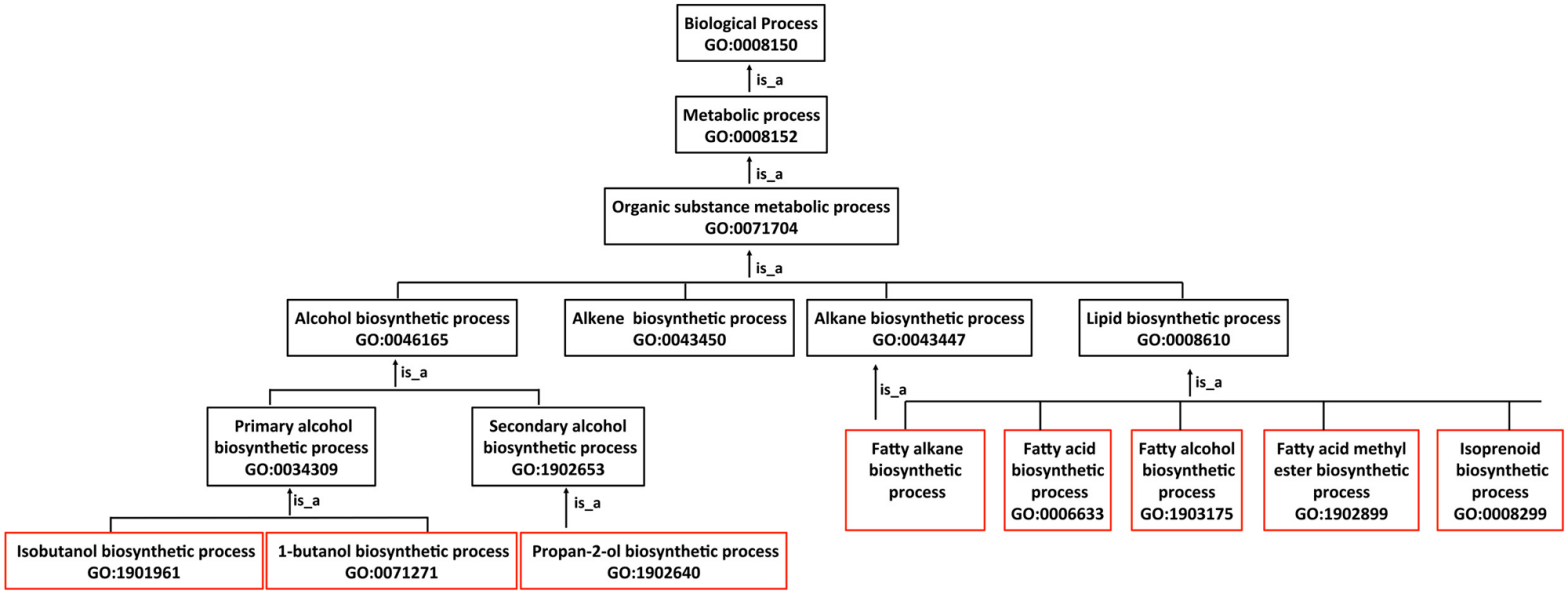
**Description of advanced biofuels using biological process terms in the Gene Ontology graph.** The GO has three ontologies, Molecular Function, Biological Process and Cellular Component, which are made up of terms (otherwise known as classes) arranged in the form of a graph. Each graph consists of a set of more specific subclasses (child) terms that form relationships (in this case “is_a”) with more general (parent) terms. As an example, biosynthesis terms for fuel and fuel-like products boxed in red are subclasses of the more general terms boxed in black.

The GO is a structured, species-neutral ontology that describes the attributes of gene products. The GO consists of three distinct ontologies: “GO:0003674 molecular function,” “GO:0008150 biological process” and “GO:0005575 cellular component,” which are made up of terms (otherwise known as classes) arranged in a graph structure. Terms in the graph are related to one another via relationships, which are of a given type. Examples of relations used in GO include “is_a,” “part_of,” “regulates,” “has_part” and “occurs_in.” Some relationships, namely “is_a” (is a type of) and “part_of,” are taxonomic such that “child terms” are more specific than the more general “parent” terms (Ashburner et al., 2000; Harris et al., 2004; **Figure 2**). Terms can have one or more “parent terms” and gene products annotated with specific child terms are automatically associated with the corresponding “parent terms” in the graph. Currently, GO uses the CHEBI database^2^ as the source of all primary chemical names (Hill et al., 2013). Alternative names are added as synonyms. We made several additions to the CHEBI as it lacked most of the chemical entities used by the MENGO consortium. The GO also actively interacts with and maintains mappings to EC, MetaCyc, Rhea, Reactome, and several other systems^3^. GO is working with Rhea and Reactome on a system of automatic import for enzymatic reactions to avoid duplication of curation effort and to further improve interoperability.

The GO is widely recognized as a powerful tool for the annotation of gene products of all organisms including important biofuel producers like *Saccharomyces cerevisiae* and cyanobacteria (Christie et al., 2009; Beck et al., 2012). The ongoing maintenance and expansion of the GO results from international collaborative efforts that are managed by the Gene Ontology consortium (GOC). The GOC encourages and works with associated groups, such as MENGO, on term development and annotation in focused subject areas.

The MENGO team has produced over 600 bioenergy-related terms^4^^,^^5^ . Included in this set are some key terms relevant to the description of advanced biofuel production. For example, terms for the microbial production of isobutanol “GO:1901961 isobutanol biosynthetic process,” isopropanol “GO:1902640 propan-2-ol biosynthetic process” and isopentenol “GO:1902934 isopentenol biosynthetic process” were additions made by the MENGO group. When specific gene products are annotated using GO terms, the information is stored in tabular form as “gene association” statements^6^, which are consolidated into a central GO database to enable public access to the annotations (Christie et al., 2009; Beck et al., 2012). Information stored in a gene association statement includes the gene product ID, the GO terms specifying the function of the gene product, its biological context and/or the location of activity. Experimental or bioinformatics evidence is represented by terms from the Evidence Code Ontology^7^ (ECO). Also included is the taxonomic source of the gene product and the reference(s) (often a PubMed identity number) indicating the publication(s) from which information was obtained. Selected gene products, annotated with GO bioenergy related terms are provided in **Table 1** and Supplementary Table 1. All other bioenergy related annotations made by MENGO can be found at the MENGO website^8^ or the GO website^9^ (AMIGO).

**Table 1:** Abbreviated Gene Ontology table showing annotations of selected experimentally characterized gene products associated with advanced biofuel production.

Gene product	Ontology*	GO term	Evidence†	Taxon	Reference
**Butanol biosynthetic process**
GO:0071271					
Thl	F	GO:0003985: acetyl-CoA acetyltransferase activity	IDA	1488	PMID:16347774
Hbd	F	GO:0008691: 3-hydroxybutyryl-CoA dehydrogenase activity	IDA	1520	PMID:1444364
Crt	F	GO:0003859: 3-hydroxybutyryl-CoA dehydratase activity	IDA	1488	PMID:16346566
Bcd	F	GO:0004085: butyryl-CoA dehydrogenase	IDA	1488	PMID:8655474
BYDH	F	GO:0004029: aldehyde dehydrogenase (NAD) activity	IDA	1488	PMID:8300540
BDH	F	GO:0004022: alcohol dehydrogenase activity	IDA	272562	PMID:11790753
**Isobutanol biosynthetic process**
GO:1901961					
Bat1	F	GO:0004084: branched-chain aminotransferase activity	IDA	4932	PMID:8798704
Bat1	C	GO:0005739: mitochondrion	IDA	4932	PMID:8798704
Bat2	F	GO:0004084: branched-chain aminotransferase	IDA	4932	PMID:8798704
Bat2	C	GO:0005829: cytosol	IDA	4932	PMID:8798704
PDC	F	GO:0004737: pyruvate decarboxylase	IMP	4932	PMID:9546164
ADH2	F	GO:0004022: alcohol dehydrogenase activity	IMP	4932	PMID:3546317
**Isopropanol biosynthetic process**
GO:1902639					
THL	F	GO:0003985: acetyl-CoA acetyltransferase activity	IDA	1488	PMID:16347774
CTF	F	GO:0008410: CoA-transferase activity	IDA	1488	PMID:2719476
ADC	F	GO:0047602: Acetoacetate decarboxylase activity	IDA	272562	PMID:2268159
SADH	F	GO:0004022: alcohol dehydrogenase activity	IDA	1520	PMID:8349550
**Tolerance**
ARI1	P	GO:1990370: process resulting in tolerance to aldehyde			
Spo0A	P	GO:1990336: process resulting in tolerance to butanol	IDA	1488	PMID:15028679
Cfa	P	GO:1990336: process resulting in tolerance response to butanol	IDA	1350	PMID:24014527
Cfa	P	GO:1990337: process resulting in tolerance to isobutanol	IDA	1350	PMID:24014527
Sll0690	P	GO:1990336: process resulting in tolerance to butanol	IMP	1148	PMID:23883549
Slr0947	P	GO:1990336: process resulting in tolerance to butanol	IMP	1148	PMID:23883549
slr1295	P	GO:1990336: process resulting in tolerance to butanol	IMP	1148	PMID:23883549

^*^Molecular function (F), biological process (P), cellular component (C).

*^†^Inferred from direct assay (IDA), inferred from mutant phenotype (IMP).*

The GO has also been used extensively in the analysis of high-throughput data including genomic, transcriptomic, proteomic, and metagenomic data (Marinković et al., 2012; Molnar et al., 2012; Benso et al., 2013; Plewniak et al., 2013; Schmidt et al., 2014). Currently, the GO is limited to terms for describing natural processes (Blake and Harris, 2008). It does not provide terms for non-natural processes such as disease states, nor does it provide terms for novel biological functions that have been produced by genetic manipulations or synthetic biology. This is a current limitation of the GO in the context of bioenergy research. The MENGO team is currently discussing with the community the creation of a complementary set of GO-like terms suitable for synthetic biological functions (which we provisionally call SYNGO). To underscore the anticipated broad scope of SYNGO, at the end of this review we provide brief descriptions of some synthetic processes and cell parts that are driving the development of this resource.

## MULTIENZYME COMPLEXES FOR DECONSTRUCTION OF BIOMASS: CELLULOSOMES AND XYLANOSOMES

Complete hydrolysis of cellulose to glucose requires the synergistic action of three general types of glycoside hydrolases (Doi, 2008; Gomez del Pulgar and Saadeddin, 2014): (i) Cellulases or endo-1,4-β-glucanases randomly hydrolyzes internal bonds in a cellulose chain releasing products of varying chain lengths. Non-processive cellulases are truly random and processive cellulases, upon binding a cellulose chain continue cutting through the bound substrate. (ii) Exo-1,4-β-glucanases “GO:0031217 glucan 1,4-beta-glucosidase activity” works from either the reducing or non-reducing end of the cellulose polymer to release cellobiose. They are sometimes called exocellulases and their processive forms are known as cellobiohydrolases “GO:0016162 cellulose 1,4-beta-cellobiosidase activity.” A variation of this group, glucohydrolase “GO:0080079 cellobiose glucosidase activity,” acts on the non-reducing end and releases glucose. (iii) β-glucosidases “GO:0008422 β-glucosidase activity” hydrolyze cellobiose released by other enzymes to glucose and are also known as cellobiases (Wilson, 2011, 2012). Activities of these enzymes on oligosaccharides have also been reported and their actions from the non-reducing end release glucose, endoglucanases, exoglucanases, and cellobiases (Chang et al., 2013).

The major component of hemicellulose is xylan. Two key enzymes collectively called xylanases “GO:0097599 xylanase activity” are responsible for hydrolysis of xylan. Endo-xylanase (endo-1,4-β-xylanase) “GO:0031176 endo-1,4-β-xylanase activity” acts on the homopolymeric backbone of 1,4-linked β-D-xylopyranose producing xylooligomers, and β-xylosidase (xylan-1,4-β-xylosidase) “GO:0009044 xylan-1,4-β-xylosidase activity” act on the xylooligomers releasing xylose (Biely et al., 1985; Ahmed et al., 2009). Additionally, accessory enzymes such as acetyl xylan esterases “GO:0046555 acetylxylan esterase activity” act to remove the side chain substitution along the xylan backbone (Juturu and Wu, 2013). The GO uses the CHEBI database^10^ as the source of all primary chemical names and all alternate names are listed as synonyms. These hydrolytic enzymes exist as free independent enzymes or as multienzyme complexes called cellulosomes “GO:0043263 cellulosome” and xylanosomes “GO:1990358 xylanosome” (Jiang et al., 2004, 2006). This section will focus on using the GO to describe these multienzyme complexes that mediate degradation of polysaccharides. Other aspects of deconstruction of biomass for biofuel can be found in several other reviews (Dodd and Cann, 2009; Blanch et al., 2011; Chundawat et al., 2011; Blumer-Schuette et al., 2014).

**FIGURE 3:**
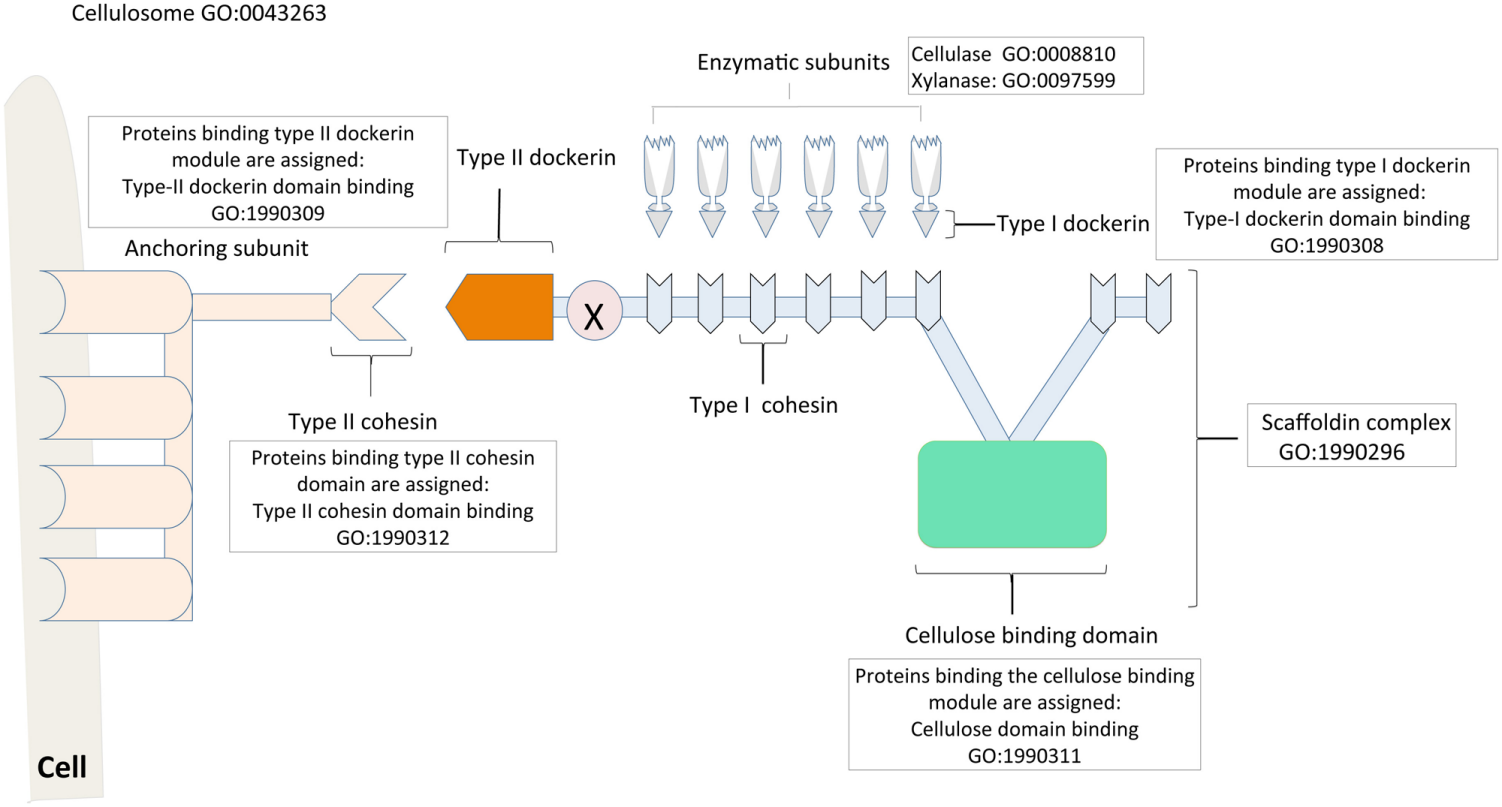
**Cellulosome assembly described with the Gene Ontology.** The cellulosome is a multi-enzyme complex involved in the deconstruction of biomass for biofuel production. The central scaffoldin subunit of the cellulosome consists of a cellulose-binding module (colored green), which binds to the cellulosic substrate; and a type-II dockerin (colored dark green), which attaches the scaffoldin to the bacterial cell surface via the type-II cohesins of the anchoring protein (colored blue). The type-I cohesins (colored yellow) of the scaffoldin in turn integrate the enzyme subunits into the cellulosome via the enzyme bearing type-I dockerin module. The different subunits of the cellulosome complex and their roles are described with appropriate Gene Ontology terms, notably the “domain binding” terms that are used to curate proteins/enzymes binding to specific modules. Relevant references are in the text. Figure is based on information provided in Smith and Bayer (2013).

The cellulosome of *Clostridium thermocellum* serve as a paradigm for this enzymatic nanomachine, and thus a model for studies of the structure and assembly process. The central component of the cellulosome is a non-catalytic “scaffoldin” subunit, which mediates a highly specific interaction between the enzyme-bearing type I dockerin modules and the resident type I cohesin modules (Bras et al., 2012; Smith and Bayer, 2013; Hong et al., 2014). The cellulose-binding domain (CBD) of the scaffoldin subunit aids in binding the complex to the cellulosic substrate. The type II dockerin module, which forms part of the primary scaffoldin in turn, binds to the type II cohesin of anchoring scaffoldins, which connect to the bacteria cell surface (**Figure 3**; Bayer et al., 1985; Bayer and Lamed, 1986). Recently, a third type of dockerin–cohesin interaction (type III) has been characterized in *Ruminoccoccus flavefaciens.* The type III dockerins and cohesins show a high degree of sequence divergence compared to their type I and II counterparts (Rincon et al., 2005; Karpol et al., 2013). Prior to our work, GO terms related to cellulosomes were “GO:0043263 cellulosome,” its regulation terms, and the different hydrolytic enzyme terms. However, the latter terms were not necessarily linked to the cellulosome term in a way that clearly indicated their associations. We used the “domain binding” aspect of the GO to make the multiple functions of the scaffoldin more obvious. To start with, the ability of the non-enzymatic scaffoldin to bind to several cellulose degradative enzymes via the cohesin domain makes it a complex. Therefore we introduced the term “GO:1990296 scaffoldin complex” which has a “part of” relationship to “GO:0043263 cellulosome.” In addition, domain-specific terms were also developed, namely: “GO:1990311 type I cohesin domain binding,” “GO:1990308 type I dockerin domain binding,” “GO:1990312 type II cohesin domain binding,” “GO:1990309 type II dockerin domain binding,” “GO:1990313 type III cohesin domain binding” and “GO:1990310 type III dockerin domain binding.” Gene products binding to each of these modules were assigned the appropriate domain binding term.

The xylanosome occurs naturally in anaerobic fungi and bacteria. However, besides containing several hemicellulases, not much is known about its assembly. We introduced the term “GO:1990358 xylanosome” into the GO. A modular nature, if any, has not been described for the xylanosome. Therefore for now, the only way to identify components of the xylanosome will be at the annotation level where the hemicellulases are annotated with their unique enzymatic activity terms using the Molecular Function ontology and also with the cellular component term “GO:1990358 xylanosome” where there is experimental evidence of an association with the xylanosome.

The synergy and combined action of hemicellulases and cellulases within these multi-enzyme systems makes them efficient in the degradation of biomass (Morais et al., 2010). Systematic annotation of the ever-increasing repertoire of dockerin–cohesin pairs of the cellulosome, using the GO terms described above, will serve to inform their use in engineering more efficient cellulosomes, xylanosomes, and cellulosome-like complexes.

## PRODUCTION OF ISOPROPANOL AND BUTANOL

**FIGURE 4:**
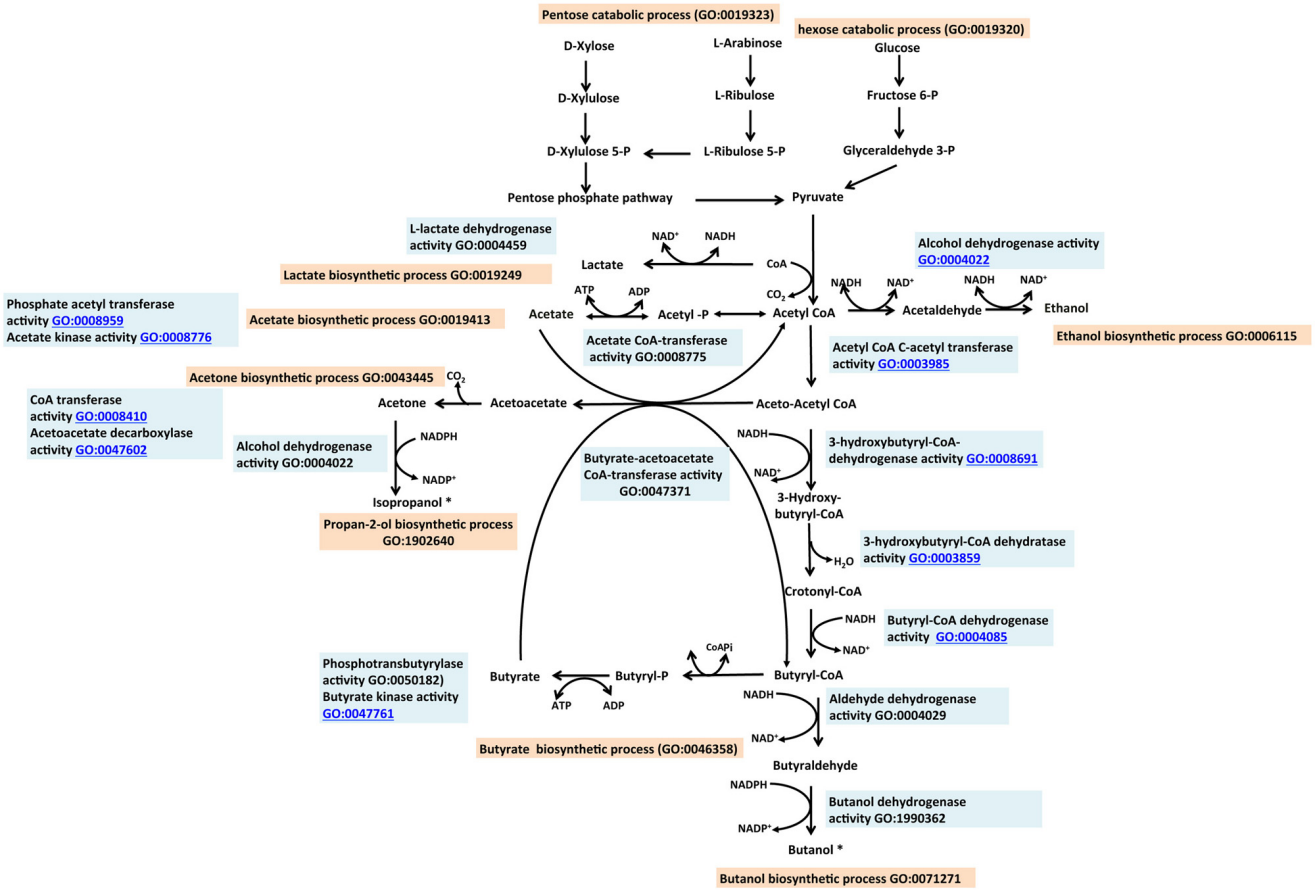
**The isopropanol, butanol ethanol (IBE) pathway described with the Gene Ontology.** Butanol and propan-2-ol (asterisked) designated as advanced biofuels are naturally produced by the IBE pathway. Role of enzymes are labeled with appropriate GO Molecular Function term (sky blue). Processes leading to the production of each alcohol are designated with appropriate GO Biological Process term (tan). Relevant references are in the text. Figure is based on information provided in Lee et al. (2008b).

Clostridia can produce isopropanol and butanol from sugars via pyruvate and acetyl-CoA [for a review of early work in this area see Stephenson (1949) and references therein]. Short chain alcohols proposed as advanced alcohols include those with C3–C4 units such as isopropanol and butanol (Gronenberg et al., 2013; Xue et al., 2013). These alcohols have superior properties relative to ethanol in terms of energy density or energy content, and ease of storage and distribution. Similar to ethanol, these can also be produced from renewable non-food feedstocks. Traditionally, butanol and isopropanol are produced as a mixture by some clostridial species such as *Clostridium acetobutylicum* possessing the acetone/isopropanol butanol–ethanol (A/IBE) pathway (**Figure 4**). Following breakdown of the pentose sugars via the pentose phosphate pathway “GO:0019323 pentose catabolic process” and hexose sugars via glycolysis, “GO:0006096 glycolysis” to pyruvate, the A/IBE process can be divided into two distinct metabolic phases: one phase involves the formation of the organic acids, butyrate and acetate (called “acidogenesis”). In the second phase, these organic acids may act as substrates for the biosynthesis of acetone and alcohols (butanol and ethanol). The latter phase is referred to as “solventogenesis.” The production of butyrate and acetate are represented in the GO as “GO:0046358 butyrate biosynthetic process” and “GO:0019413 acetate biosynthetic process,” respectively. These two child terms fall under the parent term “GO:0016053 organic acid biosynthetic process.” Solventogenic enzymes and gene products associated with the formation of acetone, butanol, ethanol, and isopropanol are annotated with appropriate GO molecular function terms and also with the biological process terms “GO:0043445 acetone biosynthetic process,” “GO:0071271 1-butanol biosynthetic process,” “GO:0006115 ethanol biosynthetic process” and “GO:1902640 isopropanol biosynthetic process,” respectively. The processes represented by these terms fall under “GO:0042181 ketone biosynthetic process” and “GO:0046165 alcohol biosynthetic process.” The terms for organic acid and solvent productions have synonyms, “acidogenesis” and “solventogenesis,” respectively. The addition of synonyms is a feature of the GO that facilitates easy access to concepts represented by diverse descriptions in the literature.

In *Clostridium*, the physiological transition from acidogenesis to solventogenesis is associated with the stationary phase of the organism’s growth cycle. The *Clostridium* Spo0A gene product has been shown to regulate solventogenesis (Ravagnani et al., 2000; Harris et al., 2002; Dürre and Hollergschwandner, 2004; Hu et al., 2011) and activate the downstream genes of the acidogenic process, in addition to controlling sporulation “GO:0043934 sporulation.” We annotated Spo0A with the term “GO:1902930 regulation of alcohol biosynthetic process” which has “regulation of solventogenesis” as a synonym, as well as with the term “GO:0043937 regulation of sporulation.” Another gene product, SpoIIE was shown to control sporulation in *C. acetobutylicum* but was not associated with solvent synthesis (Scotcher and Bennett, 2005; Bi et al., 2011). The multiple roles of Spo0A are thus documented as separate entries in the GO association table with its role in sporulation overlapping that of SpoIIE. Biosynthesis of butanol and isopropanol share the same metabolic pathway from pyruvate to acetyl-CoA and thereafter follow respective branches (**Figure 4**).

Microbial production of butanol occurs naturally in certain *Clostridium* species as part of the acetone butanol ethanol (ABE) pathway (Lee et al., 2008a; Qureshi et al., 2010). Some of these bacteria can use both pentose and hexose sugars as substrates. Typically, the breakdown of pentose and hexose sugars to pyruvate follows different paths but subsequent steps from pyruvate to butanol production may comprise the same reactions. The GO terms “GO:1990284 hexose catabolic process to 1-butanol” and “GO:1990290 pentose catabolic process to 1-butanol” are child terms of “GO:0071271 1-butanol biosynthetic process.” In the so-called CoA-dependent pathway for butanol production, the generation of butanol is initiated with two molecules of acetyl-CoA. Six enzymes encoded by seven genes mediate reactions leading to the production of butanol. Two molecules of acetyl-CoA are condensed, reduced, and dehydrated to form crotonyl-CoA, which is then reduced to butanol. The enzymes responsible for these reactions are shown in **Figure 4** and **Table 1**, with appropriate GO annotations. The key enzymes leading to butanol production are each described with a distinct molecular function term. The transition from acidogenesis to solventogenesis in some *Clostridium* species occurs at low pH as a result of acid accumulation. Butyrate, produced during acidogenesis, becomes a substrate for butanol production in the solventogenesis phase. This process is mediated by the enzymes butyrate acetoacetate CoA-transferase “GO:0047371 butyrate acetoacetate CoA-transferase activity,” butyraldehyde dehydrogenase and butanol dehydrogenase “GO:1990362 butanol dehydrogenase activity.”

Isopropanol is naturally produced by *Clostridium beijerinckii* and *Clostridium aurantibutyricum* (George et al., 1983), although the natural yield is very low (Chen and Hiu, 1986). It can be used directly as an additive to gasoline or as a feedstock for biodiesel production (Lee et al., 1995); in the latter case esterification with isopropanol is used to reduce the chances of crystallization at low temperatures. The native pathway involves the condensation of two molecules of acetyl-CoA into a molecule of acetoacetyl-CoA; the coenzyme A is then transferred to acetate or butyrate, catalyzed by CoA transferase “GO:0008410 CoA-transferase activity.” The two child terms of CoA transferase are “GO:0008775 acetate CoA transferase” and “GO:0047371 butyrate-acetoacetate CoA-transferase.” A reaction catalyzed by acetoacetate decarboxylase “GO:0047602 acetoacetate decarboxylase activity” converts acetoacetate to acetone. Subsequently acetone is converted to isopropanol in a NADPH-dependent reaction catalyzed by a secondary alcohol dehydrogenase (ADH) “GO:0050009 isopropanol dehydrogenase activity.”

## ISOBUTANOL PRODUCTION FROM VALINE AND GLYCINE

The branched-chain alcohol isobutanol, (2-methylpropan-1-ol) exhibits superior physiochemical properties similar to butanol. Isobutanol is naturally produced during fermentation by *S. cerevisiae* albeit in low amounts (Dickinson et al., 1998; Branduardi et al., 2013). Dickinson and coworkers examined the metabolic pathways used in the degradation of valine to isobutanol. Catalytic breakdown of valine to isobutanol is mediated by the Ehrlich pathway, which starts with transamination of valine to α-ketovalerate via the branched chain amino acid aminotransferase, Bat2, which is also known as Twt2p and Eca40p. The alternate names are curated in the GO association table under “database object symbol synonyms.” The location of activity of gene products is described with the GO Cellular Component ontology. Bat2 has been shown to be localized in the cytosol “GO:0005829 cytosol” and a homolog Bat1 is localized to the mitochondrial matrix “GO:0005759 mitochondrial matrix,” both having the same molecular function “GO:0004084 branched chain amino acid aminotransferase” (**Table 1**). The subsequent decarboxylation step resulting in the formation of isobutyraldehyde is mediated by ketoacid decarboxylase. All of the three isozymes of pyruvate decarboxylase (Pdc1p, Pdc5p and Pdc6p) have been shown to be capable of decarboxylating α-ketovalerate (Dickinson et al., 1998). Finally a reduction step mediated by ADH converts the aldehyde to isobutanol (Dickinson et al., 1998). The PDCs are each assigned the same GO Molecular Function terms “GO:0004737 pyruvate decarboxylase activity” in separate entries in the GO association table and the aldehyde dehydrogenase gene is assigned “GO:0004022 ADH activity.” All these gene products are also associated with the biological process term “GO:1901961 isobutanol biosynthetic process.”

Another pathway was recently discovered in *S. cerevisiae* for *de novo* isobutanol biosynthesis. A study by Villas-Bôas et al. (2005) suggested that glycine deamination led to the generation of glyoxylate and subsequently the formation of α-ketovalerate and α-isoketovalerate. Based on the above work, Branduardi et al. (2013) conducted a study to decipher the components of the pathways leading to the formation of butanol and isobutanol with glycine as substrate. Briefly, glycine is converted into serine through serine hydroxymethyltransferase (Shm2; McNeil et al., 1994), which is deaminated by serine deaminase CHA1 to form pyruvate. Pyruvate is then converted to isoketovalerate and then to isobutanol following the reactions as described above. These findings emphasize that it is important to define formation of a product based on the substrate utilized, as sometimes the same end is reached from multiple starting points. The terms “GO:1902697 valine catabolic process to isobutanol” and “glycine catabolic process to isobutanol” can appropriately capture this difference and these two terms are linked to the parent term “GO:1901961 isobutanol biosynthetic process.” The pathway also produces butanol, but since a high level of isobutanol production was observed, McNeil et al. (1994) hypothesized that carbon flux was being shunted away from butanol production toward isobutanol production by the isomerization of α-ketovalerate into α-isoketovalerate. Analogous to these natural processes in *S. cerevisiae*, Atsumi et al. (2008) employed a metabolic engineering approach, which involved diverting 2-keto acid intermediates from the amino acid biosynthetic pathway of *Escherichia coli* to produce other branched-chain alcohols such as 2-methyl-1-butanol, 3-methyl-1-butanol and 2-phenylethanol.

## FATTY ACID BIOSYNTHESIS AND FATTY ACID-DERIVED BIOFUELS

Fatty acid metabolism has attracted the most attention as a biological route to convert sugars to liquid transportation fuels (Zhou and Zhao, 2011; Lennen and Pfleger, 2013; Janssen and Steinbuchel, 2014). It is particularly suited to provide precursors for advanced biofuel because of its high efficiency and the high-energy content of the end product (Atsumi and Liao, 2008; Rude and Schirmer, 2009; Peralta-Yahya and Keasling, 2010; Wen et al., 2013). Additionally, natural metabolic pathways have been identified that convert these precursors into the biofuel product. Generally, the first committed step in fatty acid biosynthesis in *E. coli* is the conversion of acetyl-CoA to malonyl-CoA catalyzed by acetyl-CoA carboxylase (AccABCD) “GO:0003989 acetyl-CoA carboxylase activity,” which is a protein complex (GO:0009317 acetyl CoA carboxylase complex) comprising four subunits (Li and Cronan, 1992). Malonyl-CoA is then transferred to acyl carrier protein (ACP) via a malonyl-CoA:ACP transacylase (FabD, GO:0004314 malonyl-CoA:ACP transacylase activity; Campbell and Cronan, 2001; Oefner et al., 2006). Subsequently cycles of fatty acid elongation are initiated by malonyl-ACP and acetyl CoA catalyzed by β- ketoacyl-ACP synthase III (FabH, GO:0033818 beta-ketoacyl-ACP synthase III activity; Lai and Cronan, 2003). FabB and FabF use acyl-ACP as substrates to initiate successive chain elongation reactions (Feng and Cronan, 2009). A β-hydroxyacyl-ACP is the product of the second step in elongation, generated by β–ketoacyl ACP reductase (FabG, GO:0004316 beta-ketoacyl ACP reductase activity) while expending one molecule of NADPH. FabA and FabZ catalyze formation of an enoyl-ACP (Mohan et al., 1994). Following enoyl-ACP formation, the last intermediate in the fatty acid elongation cycle, acyl-ACP is formed by FabI with the consumption of NADPH (Heath and Rock, 1995). Two thioesterases (TEs) in *E. coli*, TesA and TesB release the fatty acid chains from the ACP to produce free fatty acids (Lee et al., 2006). In the biofuel field there are preferences for fatty acids of various chain lengths (Lennen and Pfleger, 2012; Torella et al., 2013). For example, longer chain products (C_12-_C_20_) fall within the diesel range, which have high energy densities (Wen et al., 2013). The GO has terms describing the synthesis of fatty acids of different chain lengths: for short-chain fatty acids, “GO:0051790 short-chain fatty acid biosynthetic process”; for medium-chain fatty acids, “GO:0051792 medium-chain fatty acid biosynthetic process”; for long-chain fatty acids “GO:0042759 long-chain fatty acid biosynthetic process”; and for very long-chain fatty acids, “GO:0042761 very long-chain fatty acid biosynthetic process.” While free fatty acids are valuable, they cannot be used directly as fuels and must first be converted either to fatty acid alkyl esters (for biodiesel), or to fatty acid-derived alkanes, alkenes or fatty alcohols (Zhang et al., 2011; Peralta-Yahya et al., 2012; Wen et al., 2013).

### FATTY ACID-DERIVED ALKANES AND ALKENES

**FIGURE 5:**
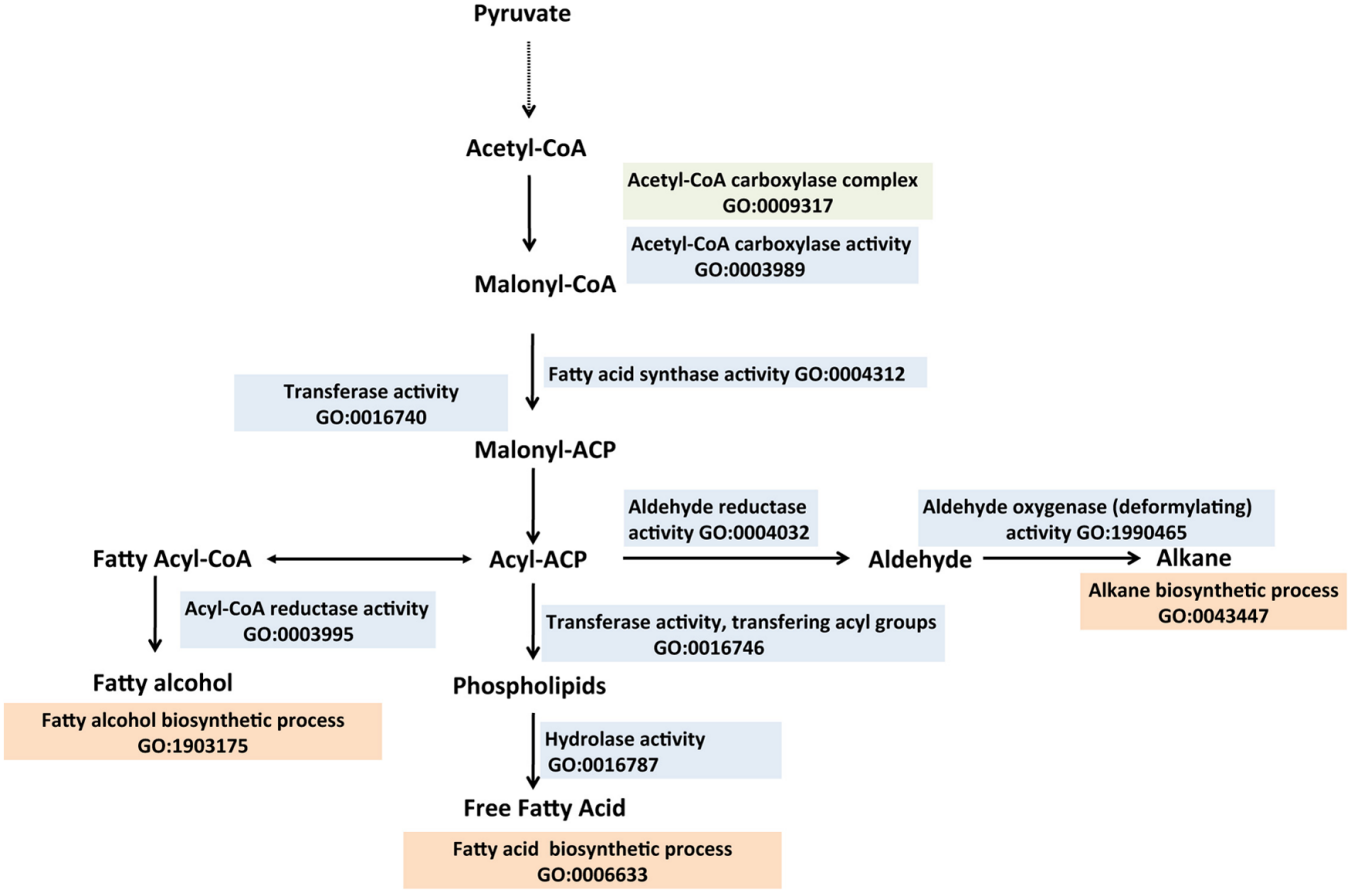
**Biosynthesis of fatty acids, fatty alkanes and fatty alcohols described with the Gene Ontology.** Fatty acids are generated from acetyl-CoA and malonyl-CoA precursors through fatty acid synthases. Acyl-ACPs, which are intermediates of the fatty acid biosynthetic process, are reduced to fatty alcohols or deformylated to form alkanes. Enzymes involved in the biosynthesis of the fatty acid derivatives are described with appropriate GO Molecular Function terms (colored sky blue), Biological Process terms (tan) and the Cellular Component terms (green). Relevant references are in the text. Figure is based on information provided in Lennen and Pfleger (2013).

Alkanes, an integral part of fossil fuels (gasoline, diesel and jet fuel), are naturally found in diverse organisms including plants, insects and microbial species, but the genetic and biochemical bases behind the production of alkanes have been elusive. Those with C4–C23 carbon chain length possess higher energy densities, hydrophobic properties and compatibilities with existing liquid fuel infrastructure. Most evidence supporting the decarbonylation of aldehydes (a fatty acid metabolite) as the primary mechanism for alkane production have been obtained in eukaryotic systems (Cheesbrough and Kolattukudy, 1984; Dennis and Kolattukudy, 1991, 1992). This in part informed the identification of the *Arabidopsis cer* gene as encoding a protein with decarbonylase activity (GO:0071771 decarbonylase activity) involved in alkane biosynthesis. (Aarts et al., 1995). It is only recently that the pathway of alkane biosynthesis was elucidated in cyanobacteria (**Figure 5**; Schirmer et al., 2010; Li et al., 2011, 2012) making this class of hydrocarbons eligible to be categorized as a next generation biofuel (advanced). In this pathway, fatty acids are reduced by a two-step reaction to alkanes. Fatty acyl-ACP (fatty acid metabolite) is reduced to a fatty aldehyde via a fatty acyl-ACP reductase. This is then followed by a deformylation step catalyzed by an aldehyde deformylating oxidase “GO:1990465 aldehyde oxygenase (deformylating) activity,” resulting in alkane production (Schirmer et al., 2010; Li et al., 2011, 2012; Warui et al., 2011; Coates et al., 2014). Commonly found hydrocarbons in cyanobacteria are heptadecane (GO:1900636 heptadecane biosynthetic process) and methyl heptadecane (Schirmer et al., 2010; Zhang et al., 2011), which have cetane numbers of 105 and 66 respectively (requirement for US diesel or ASTM standard, 47 minimum (Rashid et al., 2008) making the hydrocarbon products from cyanobacteria ideal candidates for diesel fuel applications.

Another group of hydrocarbons produced from fatty acid derivatives are olefins (alkenes, GO:0043450 alkene biosynthetic process). In *Jeotgalicoccus* sp., the terminal alkenes, 8-methyl-1-nonadecene and 17-methyl-1-nonadecene have been identified. A terminal olefin-forming fatty acid decarboxylase belonging to the cytochrome P450 family of enzymes (OleT) has been identified as the key enzyme involved in the production of these olefins in *Jeotgalicoccus* sp. (Rude et al., 2011). Other studies proposed a head-to-head condensation of fatty acids, which involves the formation of a carbon-to-carbon bond between the carboxyl carbon of one fatty acid and the α-carbon of another fatty acid as another mechanism to generate long chain (C_23_–C_33_) olefins. OleA, a homolog of the condensing enzyme in fatty acid biosynthesis (FabH -3-oxo-acyl-ACP ketosynthase (KS) III), was identified as a key enzyme in long chain olefin production (Beller et al., 2010; Sukovich et al., 2010). In cyanobacteria, the olefin-producing pathway (OLS) involves a polyketide synthase (GO:0034081 polyketide synthase complex) that first elongates fatty acyl-CoA with two carbons from malonyl-CoA via KS and acyl transferase (AT) domains. This is followed by reduction to the hydroxyacid by a ketoreductase (KR, GO:0045703 ketoreductase activity; Mendez-Perez et al., 2011). In the final step, sulfotransferases (ST, GO:0008146 sulfotransferase activity) activate the β-hydroxy group via sulfonation, and then a TE acts on this substrate to catalyze decarboxylation and loss of sulfate to form the terminal alkene (McCarthy et al., 2012).

### FATTY ACID ALKYL ESTERS (BIODIESEL)

Biodiesel is a substitute for petroleum-based diesel fuel. Like its counterparts described above, biodiesel has properties similar to those of diesel, and therefore, can be used in the diesel engines and stored and distributed using the existing infrastructure. Other advantages of biodiesel include reduced fuel toxicity and increased lubricity. Additionally the use of biodiesel leads to lower carbon monoxide and soot emissions than conventional diesel fuels. Biodiesel is traditionally produced by the trans-esterification of mostly plant-derived triacylglycerols yielding glycerol and fatty acid alkyl esters (FAAE), particularly fatty acid methyl esters (FAMEs; “GO:1902899 FAME biosynthetic process”). However, biodiesel production is limited by the availability of inexpensive vegetable oil feedstocks. This has prompted a search for sustainable alternatives. Direct microbial production of FAAEs is an area of intense research as it bypasses the transesterification step, reducing cost and energy and also avoids the use of methanol, (Kalscheuer et al., 2006; Nawabi et al., 2011) which is an expensive and toxic feedstock.

## ISOPRENOID-DERIVED BIOFUEL

**FIGURE 6:**
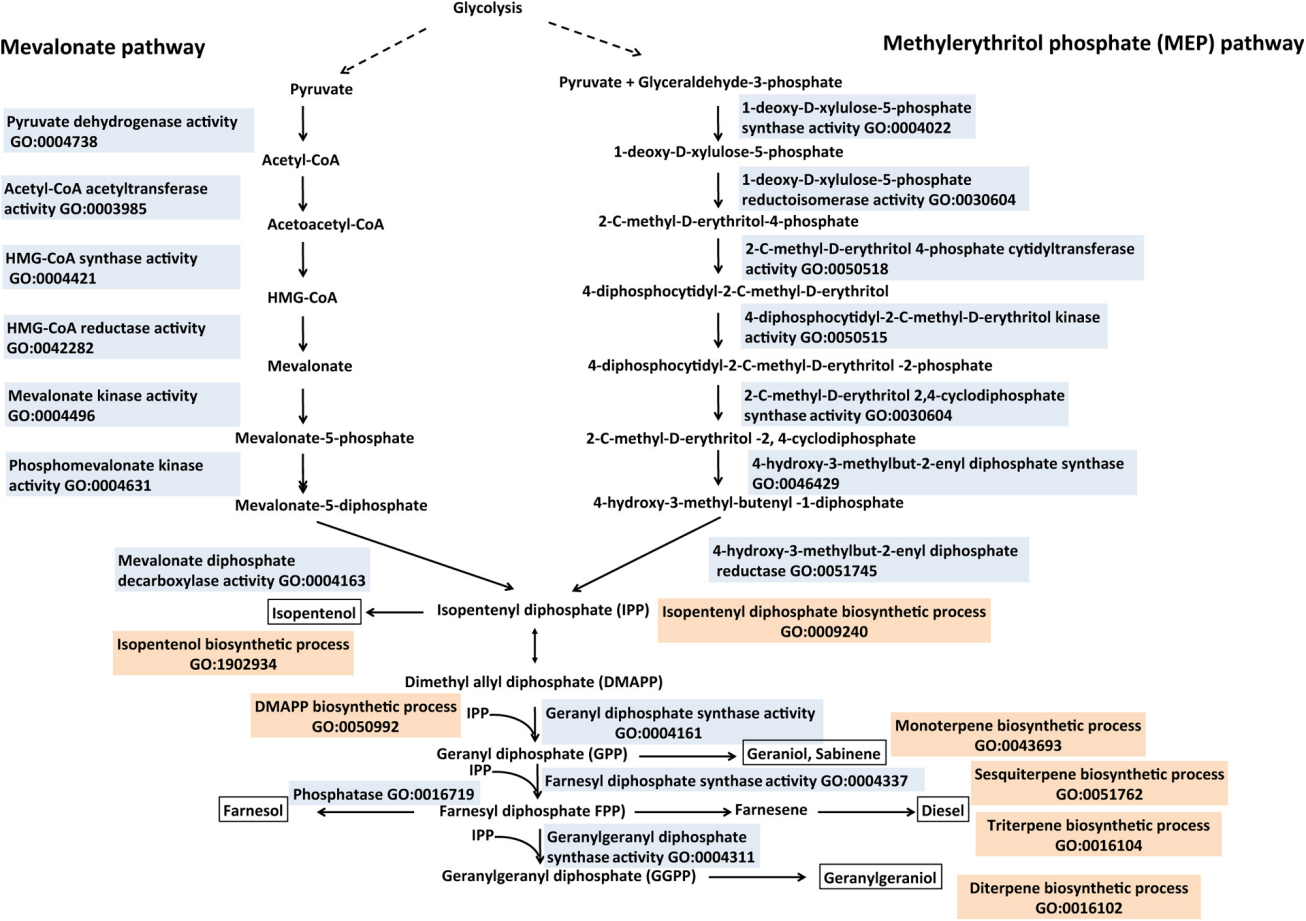
**Biosynthesis of isoprenoid-derived biofuels described with the Gene Ontology.** Isoprenoids are synthesized from two isomeric 5-carbon unit compounds called IPP (isopentenyl pyrophosphate) and DMAPP (dimethyl-allyl pyrophosphate). Both isoprenoid precursors are formed from the mevalonate (MEV) or the 1-deoxy-D-xylulose-5-phpsphate (DXP) pathways. IPP and DMAP can be further converted to prenyl-pyrophosphates like geranyl diphosphate (GPP), farnesyl diphosphate (FPP), and geranylgeranyl diphosphate (GGPP) via prenyltransferases. IPP and corresponding prenyl pyrophosphates have been exploited to produce desired biofuels (boxed) such as isopentenol, geraniol, and farnesol. Enzymes involved in isoprenoid biosynthesis are described with appropriate GO Molecular Function terms (colored in sky blue), the Biological Process terms are in tan color. Relevant references are in the text. Figure is based on information provided in Peralta-Yahya and Keasling (2010) and Yikmis and Steinbuchel (2012).

Isoprenoids, also called terpenes, have been evaluated for pharmaceutical, nutritional and fuel products (Bohlmann and Keeling, 2008; Peralta-Yahya and Keasling, 2010). Two independent biosynthetic pathways for isoprenoid production “GO:0008299 isoprenoid biosynthetic process” are found in nature (**Figure 6**). The methylerythritol phosphate (MEP) pathway “GO:1902768 isoprenoid biosynthetic process via 1-deoxy-D-xylulose 5-phosphate” and the mevalonate pathway “GO:1902767 isoprenoid biosynthetic process via mevalonate” have evolved for the production of the key five-carbon isoprenoid intermediates, isopentenyl diphosphate (IPP), and dimethylallyl diphosphate (DMAPP; Beytia and Porter, 1976; Edwards et al., 1992; **Figure 6**). The MEP pathway consists of seven steps resulting in the conversion of glyceraldehyde-3-phosphate and pyruvate to IPP and DMAPP and it is found in most bacteria, chloroplast, unicellular eukaryotes, and certain parasites (Zhao et al., 2013; Jarchow-Choy et al., 2014; **Figure 6**). The mevalonate pathway is responsible for all the isoprenoid production in archaea, some bacteria and most eukaryotes (Miziorko, 2011; **Figure 6**). It converts acetyl CoA in six steps to IPP via the key intermediate mevalonate. Specifically, acetyl-CoA and acetoacetyl-CoA are condensed into 3-hydroxy-3-methylglutaryl-CoA (HMG-CoA), which is reduced to mevalonate via HMG-CoA reductase (HMGR, GO:0042282 HMG-CoA reductase activity). Following this are two phosphorylation steps, which convert mevalonate to mevalonate-5-diphosphate via the actions of mevalonic acid kinase “GO:0004496 mevalonic acid kinase activity” and phosphomevalonate kinase “GO:0004496 phosphomevalonate kinase activity.” An ATP-coupled decarboxylation step yields mevalonate-5-diphosphate to the C5 building block IPP. An IPP isomerase “GO:0004452 IPP isomerase activity” is responsible for the interconversion of IPP and DMAPP. Condensation of IPP and DMAPP using prenyltransferases “GO:0004659 prenyltransferase activity” yields several prenyl-pyrophosphates including farnesyl-pyrophosphate, geranyl-pyrophosphate and geranylgeranyl-pyrophosphate. The prenyl-pyrophosphatase in turn is converted to diverse terpenes including monoterpenes, sesquiterpenes and diterpenes via terpene synthase. The prenyl-pyrophosphates can be hydrolyzed by pyrophosphatases “GO:0016462 pyrophosphatase activity” to form fuel-like esters and alcohols such as isoamylacetate and isopentenol “GO:1902934 isopentenol biosynthetic process.” One such pyrophosphatase “GO:0016462 pyrophosphatase activity,” nudF from *B. subtilis*, was shown to produce isopentenol in *E. coli*. Curating the biosynthesis of the intermediates and the vast diversity of terpene synthases “GO:0010333 terpene synthase activity” therefore will provide a useful resource, which will inform decisions in the synthesis of terpene-based fuels. A new class of terpenes (C_35_), the sesquarterpenes has recently been classified (Sato, 2013) and it will be interesting to know if these compounds could provide precursors for terpene-based biofuels.

## POLYHYDROXYALKANOATE-DERIVED BIOFUEL

Microbially produced PHAs have attracted attention as biodegradable polyesters and quite recently as biofuels (Zhang et al., 2009). PHAs are synthesized inside cells during oxygen, phosphorous or nitrogen starvation in the presence of excess carbon and found as insoluble cytoplasmic inclusions called polyhydroxyalkanoate granules (GO:0070088 PHA granule; Pfeiffer and Jendrossek, 2012; Jendrossek and Pfeiffer, 2013). Synthesis of a PHA could occur through several metabolic pathways and from a variety of carbon precursors including glucose, glycerol, and fatty acids (Park et al., 2012; Shahid et al., 2013). PHAs comprise over 150 both homo- and hetero-polymers of which poly-3-hydroxybutyrate (poly (3HB), P (3HB) or PHB) being the most abundant naturally produced and most studied polyhydroxyalkanoate “GO:0042618 polyhydroxybutyrate biosynthetic process” (Laycock et al., 2014). On the basis of the carbon chain lengths of the monomers, PHAs are divided into two main groups: short chain length PHAs (scl-PHA) containing monomer units with 3–5 carbon atoms, and medium chain length PHAs (mcl-PHA) composed of monomer units with 6–18 carbon atoms. Based on the bacterial strains, PHA synthases and carbon sources involved, products of PHA biosynthesis can be a homopolymer, copolymer, block polymer or even blends (Lu et al., 2009). Functional groups such as unsaturated bonds, benzene, halogens and cyclic chemicals and epoxides can also modify PHA structures, opening up the potential for the production of a vast number of diverse PHAs. The biosynthesis of PHA “GO:190144 polyhydroxyalkanoate biosynthetic process” is quite complex and involves several enzymes that are directly or indirectly involved in PHA synthesis (Reddy et al., 2003; Park et al., 2012). PHA synthesis is well studied in the model organism *Ralstonia eutropha* (Riedel et al., 2014). In this organism, the carbon source is converted into coenzyme A thioesters of (R)-hydroxyalkanoic acids. Following this step, β ketothiolase “GO:0003988 acetyl-CoA C-acyltransferase activity” catalyzes the condensation of two coenzyme-A thioester monomers such as acetyl-CoA and propionyl-CoA. An (R)-specific reduction step involving acetoacetyl-CoA reductase “GO:0018454 acetoacetyl-CoA reductase activity” produces (R)-3 hydroxybutyryl-CoA or (R)-3 hydroxyvaleryl-CoA which is then converted into PHA by the action of PHA synthase. In regards to biofuels, 3-hydroxyalkanoates (3HAs; mcl PHAs) are linked by ester bonds formed with the hydroxyl group (–OH) of one monomer and the carboxyl (–COOH) group of the other monomer through the catalysis by various PHA synthases (Reddy et al., 2003). These hydroxyalkanoate esters (3HA esters) have been found to be similar to methyl esters of long chain fatty acids (in biodiesel). As such they can be used as fuel additives. Hydroxybutyrate and hydroxyalkanoate methyl esters (3HBME and 3HAME), generated from esterification of scl PHB and mcl PHA with methanol, respectively, are considered equivalent to ethanol (Gao et al., 2011). These esters can also be used as gasoline and biodiesel additive (Gao et al., 2011).

## TOLERANCE TO BIOFUELS AND BY-PRODUCTS

Accumulation of biofuels as well as by-products often affect the integrity of the fuel producing organism’s cell membrane and also impairs metabolic pathways associated with cell growth, thus compromising product titers (Heipieper et al., 2007; Segura et al., 2012). Most native producers of biofuels are sensitive to the solvents they produce. Advances are being made toward removing this block through investigations on the mechanisms of tolerance to product accumulation (McEvoy et al., 2004; Fujita et al., 2006; Liu and Qureshi, 2009a; Ghiaci et al., 2013; Zingaro and Terry Papoutsakis, 2013) and several organisms are being investigated as better choices for biofuel production from this standpoint (Dunlop, 2011). These efforts will be substantially aided by curating all gene products associated with native or improved tolerance to biofuels and their by-products. Tolerance is considered a phenotype, not a process and is therefore out of the scope for GO Biological Process ontology. To circumvent this problem, we created the terms “process resulting in tolerance to x” instead of “tolerance to x” (x is any compound). Here we present some examples.

The common inhibitors to microbial growth that are found in lignocellulose hydrolysates include aldehydes, ketones, phenols and organic acids (Klinke et al., 2004; Jonsson et al., 2013). A *S. cerevisiae* gene, *ARI1*, encoding a novel NADPH-dependent aldehyde reductase has been shown to be involved in providing tolerance to inhibitors such as furfural, vanillin and cinnamaldehyde that arise from lignocellulose hydrolysis (Liu and Moon, 2009b). We annotated the gene product ARI1 with GO terms for its molecular role “GO:0018455 aldehyde reductase (NADPH/NADH) activity” and a biological process term “GO:1990370 process resulting in tolerance to aldehyde” to describe its ability to confer tolerance to several aldehydes. Other notable *S. cerevisiae* enzymes with efficient aldehyde reduction activities include ADHs (ADH1, ADH6 and ADH7; GO:0004022 ADH activity), aldehyde dehydrogenase (ALD4; GO:0004030 aldehyde dehydrogenase activity) and methylglyoxal reductases (GRE2 and GRE3; GO:0043892 methylglyoxal reductase activity; Petersson et al., 2006; Laadan et al., 2008).

Tolerance to alcohols such as butanol in *C. acetobutylicum* is influenced by the multifunctional Spo0A protein, which, as discussed in an earlier section, controls sporulation and solventogenesis (Alsaker et al., 2004). Kanno et al. (2013) obtained several aerobic and anaerobic bacterial isolates from soil, spanning diverse genera that tolerate butanol and isobutanol levels greater than 2% (vol/vol). The *cfa* gene, which encodes cyclopropane fatty acid (CFA) synthase in one of these bacteria (belonging to the *Firmicutes* phylum), confers solvent tolerance in recombinant *E. coli* (Kanno et al., 2013). CFA is annotated with the terms “GO:1990336 process resulting in tolerance to butanol” and “GO:1990337 process resulting in tolerance to isobutanol.” A quantitative transcriptomic analysis revealed that in the cyanobacterium *Synechocystis* sp. PCC 6803, which uses solar energy and carbon dioxide as sole energy and carbon sources, over 250 genes are induced upon exposure to butanol (Zhu et al., 2013). Of these, three, *sll0690*, *slr0947,* and *slr1295* were further characterized using knock-out mutants. The results indicated butanol sensitivity in strains that lacked any of these genes, indicating their involvement in resistance to butanol. Using a genome-scale analysis in *S. cerevisiae*, Gonzàlez-Ramos et al. (2013) demonstrated the role of protein degradation in tolerance to C3 and C4 alcohols (butanol, 2-butanol, isobutanol, and isopropanol). Specifically, the YLR224W gene was found to be associated with increased butanol tolerance and it encodes a subunit of the Skp-Cullin_F-box (SCF) ubiquitin ligase that recognizes damaged proteins. The efflux pumps involved in the extrusion of toxins from cells have been considered appropriate candidates for studies on solvent tolerance (Segura et al., 2012). Such a pump consists of multiple proteins forming a multicomponent complex “GO:1990281 efflux pump complex,” which span the inner to the outer membrane of bacteria cells (Andersen, 2003; Du et al., 2014). An exposure to solvents induces the expression of s*rpABC* genes in *P. putida* (Kieboom et al., 1998b). The SrpABC share considerable sequence similarities to multi-drug efflux pump proteins (Kieboom et al., 1998a). Specifically, SrpA functions as a periplasmic linker protein, SrpB as an inner membrane transporter, and SrpC, as an outer membrane channel. For these reasons, besides annotations for their roles in efflux pump and cellular locations (“GO:0015562 efflux transmembrane transporter activity” from Molecular Function ontology; “GO:1990281 efflux pump complex,” “GO:0042597 periplasmic space,” “GO:0009276 Gram-negative-bacterium type cell wall,” and “GO:0019867 outer membrane from the Cellular Component ontology”), we have qualified these proteins with a biological process term “GO:1990367 process resulting in tolerance to organic substance.” Rojas et al. (2001) have identified three different solvent efflux pumps in *P. putida*, TtgABC, TtgDEF, and TtgGHI, which extrude toluene. TtgABC also could extrude antibiotics (Ramos et al., 1998; Mosqueda and Ramos, 2000; Rojas et al., 2001). All these multi-protein efflux pumps are assigned the GO term “GO:1990281 efflux pump complex.” Hydrocarbons including nonane, decane, and undecane were shown to be toxic to *S. cerevisiae* when these solvents accumulated inside the cell. A transcriptomic analysis identified modified cell membrane and efflux pumps as contributing to alkane export and tolerance. Specifically efflux pumps Snq2p and Pdr5 were shown to reduce intracellular levels of decane and undecane thereby enhancing tolerance to the alkanes “GO:1990373 process resulting in tolerance to alkane” (Ling et al., 2013).

## SYNTHETIC BIOLOGY

The yields of most fuels or fuel derivatives in the native biological systems discussed above are insufficient to be cost-competitive with petroleum-derived fuels, and therefore the technologies of metabolic engineering and synthetic biology are being employed to increase production and/or generate entirely new fuels with superior qualities. As mentioned in the introduction, currently the GO is limited to terms for describing natural processes. The MENGO team is currently discussing with the community the creation of a complementary set of GO-like terms suitable for the annotation of synthetic biological systems, which we provisionally call SYNGO. To underscore the anticipated broad scope of SYNGO, we provide here brief descriptions of selected synthetic processes and parts that are driving the development of commercially viable routes for the production of advanced biofuels.

Based on the modular nature of cellulosomes and the availability of a variety of dockerin-cohesin pairs, efforts are underway to construct designer cellulosomes, xylanosomes, and cellulosome-like structures. In such a precision-engineered multienzyme complex, the molecular architecture and enzyme content are well controlled, and the result is enhanced synergistic deconstruction of biomass (Mitsuzawa et al., 2009; Nordon et al., 2009; Smith and Bayer, 2013; Srikrishnan et al., 2013; Vazana et al., 2013). For example, Mitsuzawa et al. (2009) modified a thermostable group II chaperonin (18-subunit self-assembling protein complex called rosettasome), from the archaeon *Sulfolobus shibatae*, for use as a scaffold to assemble selected hydrolytic enzymes. A cohesin module was fused to each of the eighteen subunits which in turn was combined with dockerin-containing cellulases from *C. thermocellum* to build an 18 enzyme cellulosome-like structure they termed rosettazyme. Truncated cellulosomes also called minicellulosomes have been constructed in an effort to fully understand the relationship between cellulosome structure and enzymatic activity (Arai et al., 2007; Cha et al., 2007; Mingardon et al., 2007). Examples are the mini-CipA and mini-CipC1 cellulosomes from *Clostridium cellulovorans* and *Clostridium cellulolyticum,* respectively (Murashima et al., 2002; Perret et al., 2004). The engineered minicellulosome platforms can be extended to build complex designer cellulosomes. Designer cellulosomes are not described in the GO and a complementary term “synthetic multi-cellular complex” would be useful to capture designer multienzyme complexes such as the rosettazyme, mini-CipA and mini-CipC1 and their components.

Generally, yield is one of the focal points for improving alcohol production by means of synthetic biology technology (Peralta-Yahya et al., 2012; Gronenberg et al., 2013). For example, low butanol yield in the native Clostridium species is not economically competitive, and thus the Clostridrial CoA-dependent pathway has been heterologously expressed in genetically tractable industrial microbes hosts such as *E. coli and S. cerevisiae* (Inui et al., 2008; Steen et al., 2008) for facile manipulation toward better yield and productivity. For example, *E. coli* strain bearing the CoA-dependent pathway was further modified by substituting the reversible, flavin-dependent butyryl-CoA dehydrogenase (Bcd) with an irreversible trans-enoyl-CoA reductase (Ter) for the reduction of crotonyl-CoA (Bond-Watts et al., 2011). The increased flux to butanol improved product yield (Bond-Watts et al., 2011). While Bcd is associated with “butanol biosynthetic process” in the GO, Ter is not, as it is part of a non-natural (synthetic) pathway. This limitation in the GO could potentially be addressed with complementary SYNGO terms, for example, Ter could be assigned “enoyl-CoA reductase involved in increased butanol biosynthesis.”

The natural ability of *E. coli* to produce fatty acids and creation of new biochemical reactions through synthetic biology have provided the means to divert fatty acid metabolism toward the production of fuels (Clomburg and Gonzalez, 2010; Wen et al., 2013). This approach is a more sustainable alternative than, for example, the production of biodiesel from plant oils. Direct microbial production of fatty acid esters (biodiesel) eliminates the need for a subsequent chemical transesterification step. A pathway which includes an acyl-CoA ligase and the broad specificity acyltransferase (WS/DGAT; AtfA), together with the enzymes that provide ethanol for esterification [pyruvate decarboxylase (pdc) and alcohol dehydrogenase B (adhB)] were incorporated into a fatty acid overproducing *E. coli* strain for the production of fatty acid ethyl esters (Stoveken et al., 2005; Steen et al., 2010). Curating the components of the engineered pathway with complementary SYNGO terms, associating them with fatty acid ethyl ester biosynthesis, should be a powerful tool for researchers working to improve these processes.

## CONCLUSION

The MENGO group has created and continues to create GO terms relevant to microbial bioenergy research. In addition, the team has used the terms to produce high quality “gold standard” annotations of gene products based on the experimental scientific literature, which are useful for engineering of microbial strains to optimize bioenergy production. These resources provide easy access to otherwise dispersed information in the scientific literature and also aids in the computational analysis of large datasets. The GO annotations can also be used to uncover metabolic pathways, and to understand bioenergy-relevant microbes at the system biology level. Community involvement in MENGO term development and annotations is welcome and will create a more comprehensive resource for use by all. Since the GO currently is restricted to natural processes, a need has arisen for a complementary set of GO-like terms to describe engineered processes and the gene products that comprise them. Community involvement in this other development is also essential.

## Conflict of Interest Statement

The authors declare that the research was conducted in the absence of any commercial or financial relationships that could be construed as a potential conflict of interest.

## Footnotes

1 http://www.mengo.biochem.vt.edu

2 http://www.ebi.ac.uk/chebi/

3 http://wiki.geneontology.org/index.php/Enzymes_and_EC_mappings

4 http://www.mengo.biochem.vt.edu/

5 http://tinyurl.com/o2l7nsy

6 http://www.geneontology.org/GO.format.annotation.shtml

7 http://www.evidenceontology.org/

8 http://www.mengo.biochem.vt.edu/

9 http://tinyurl.com/o2l7nsy

10 http://www.ebi.ac.uk/chebi/

## SUPPLEMENTARY MATERIAL

The Supplementary Material for this article can be found online at:
